# Proprioception deficits in chronic stroke—Upper extremity function and daily living

**DOI:** 10.1371/journal.pone.0195043

**Published:** 2018-03-30

**Authors:** Debbie Rand

**Affiliations:** Department of Occupational Therapy, School of Health Professions, Sackler Faculty of Medicine, Tel Aviv University, Tel Aviv, Israel; University of Chicago, UNITED STATES

## Abstract

**Background:**

Proprioception deficits are common post-stroke and predict poor functional outcome. It is unknown if the presence of proprioception deficits is negatively associated with the motor and functional ability of the affected upper extremity and daily living at the chronic stage post-stroke.

**Aims:**

1) To describe proprioception deficits of individuals with chronic stroke, 2) to correlate the severity of proprioception deficits with the motor and functional ability of the upper extremity, and 3) to compare independence in basic and instrumental activities in daily living (BADL, IADL), upper extremity motor and functional abilities between individuals with and without proprioception deficits.

**Methods:**

102 adults aged 29–85 years with chronic stroke participated in this cross sectional study. The upper extremity was assessed for proprioception (Thumb localization Test), motor [Fugl-Meyer Motor Assessment (FMA)] and functional ability [Action Research Arm Test (ARAT), Box and Block Test (BBT)], grip strength and daily use [Motor Activity Log (MAL)]. Independence in BADL and IADL was also assessed.

**Results:**

71 participants had intact proprioception, 31 participants had mild-moderate proprioception deficits. Negative significant (p<.001) correlations were found between the severity of proprioception deficits to the motor ability (FMA) (r = -.41), functional ability (ARAT) (r = -.48), dexterity (BBT) (r = -.43), grip strength (r = -.41) and daily-use (MAL amount and quality) (r = -.55 and r = -.54, respectively) of the affected upper extremity. Significant between-group differences were found for BADL, IADL and upper extremity measures.

**Conclusion:**

Proprioception deficits of individuals with chronic stroke are negatively associated with upper extremity motor and functional abilities and independence in daily living. Therefore, proprioception should be assessed at the chronic stage post-stroke.

## Introduction

Proprioception is impaired in a large percentage of individuals following stroke [1–4]. The prevalence of proprioceptive deficits in individuals with an affected upper extremity post-stroke has been reported to range between 30 to 48%. Since intact proprioception is critical for movement control and functioning [4,5], it is important to understand the role of proprioception in the recovery post-stroke. Proprioception is the perception of the position, motion, and force generated by the body [6–9]. Position perception indicates the awareness of the relative position of body parts in space. This sensory information derives from muscle spindles, Golgi tendon organs, joint and cutaneous receptors [10–13].

The presence of proprioception deficits has been found to be an important predictor of poor functional outcome post-stroke [14–16], specifically in achieving independence in basic activities of daily living (BADL) and hospital length of stay [4, 5, 17, 18]. However, limited studies have focused on the impact of proprioception deficits on the recovery of the affected upper extremity post-stroke. Affected upper extremity functional recovery was found to be significantly negatively correlated to proprioception deficits and positively correlated to the initial motor ability and mental status [19]. Shoulder pain and other upper extremity complications are common in individuals with combined motor and proprioception deficits (50%) compared to individuals with a pure motor deficit (7%) [20]. Although, the presence of proprioception deficits on admission to rehabilitation was not related to motor or functional upper extremity recovery in the first 6-weeks following stroke [21], it was found to add to the prediction of daily arm-use one-year post-stroke [22].

A recent review [23] summarized the knowledge regarding proprioception deficits following stroke, however it did not address chronic stroke, and the prevalence and impact of proprioception deficits at the chronic stage post-stroke are still unknown. This information may shed light on the associations between proprioception deficits to recovery of the affected upper extremity and daily living post-stroke. Therefore, the aims of this cross-sectional study were 1) to describe proprioception deficits of individuals with chronic stroke, 2) to correlate the severity of proprioception deficits with the motor and functional ability of the upper extremity, and 3) to compare independence in BADL and IADL, upper extremity motor and functional abilities between individuals with and without proprioception deficits.

## Materials and methods

### Population

Adults with chronic stroke who lived in the community were invited to participate in the study. Inclusion criteria were 1. sustained a stroke at least 6 months prior to the study, 2. without a significant cognitive deficit (score > 21/30 points on The Mini Mental State Examination [24], a valid and reliable screening test for cognition post-stroke), 3. able to walk at least 10-meters (with or without assistance), to increase the likelihood of independence in daily function. Participants with other neurological conditions were excluded from the study.

### Procedure

This study was approved by the Research Ethics Committee in the rehabilitation center and University institutional review board. Participants were recruited using discharge lists from a large rehabilitation center. They were contacted by phone and when found eligible, were invited to the rehabilitation center for the assessment session. First, they signed the informed consent form and then underwent the assessments. Participants received reimbursement for their travel expenses.

### Tools

The following assessments were used to assess the affected upper extremity; proprioception, motor and functional ability, grip strength and daily use. Dexterity and grip strength of the less-affected (stronger) upper extremity were assessed as well.

Proprioception was assessed using the **Thumb Localization Test (TLT)** [5, 15]. The participant’s affected arm is moved by the examiner to four different locations in space and each time, the participant is requested to find and grasp their affected thumb with their less-affected hand while their eyes are closed. The scoring for each of the four attempts is based on the estimated distance that he/she misses their affected thumb in space and discriminates between; no (precisely grasps the thumb), mild (just misses to grasp the thumb and immediately corrects), moderate (is able to find the arm and then uses the arm to find the thumb), or severe proprioception deficits (cannot locate his/her thumb) [5, 15]. The final TLT score is according to the worst performance [i.e. if on three trials the participant just misses to grasp his affected thumb (mild) but on one location, he uses his arm to find his thumb (moderate); he will be scored to have moderate proprioception deficits]. This test was reported to be acceptable clinically [25]. It is assumed that by blindfolding the participant, the visual map of position sense is abolished and success is due to the activity of the proprioception map encoding limb position relative to the body, based on the information arising from the muscle [26]. Proprioception deficits assessed by the TLT have been reported to be significantly correlated with the Finger Shift Test [27], a common test to assess proprioception (r = 0.53–0.71, p<.01) as assessed on week 0, 2, 4 and 6 weeks following admission to rehabilitation [28]. Despite this, the validity and reliability of the TLT, similar to other tests for proprioception have not been well established [25]. Neither the participants nor assessors were aware of the purpose of the study.

The motor ability of the affected upper extremity was assessed using the upper extremity subtest of the **Fugl-Meyer Motor Assessment (FMA)** [29], which is a valid and reliable tool for individuals with stroke [30–31]. Scores range from 0 points (no active movement) to 66 points (full active movement).

The functional ability of the upper extremity was assessed with the **Action Research Arm Test (ARAT)** [32], a valid and reliable measure of upper extremity function [33]. The ARAT assesses grasp, grip, pinch, and gross arm movement of the affected upper extremity while performing functional tasks of picking up and transferring objects of different size. The total scores range from 0 (a non-functional hand) to 57 points (a fully functional hand).

**The Box and Block Test** [34], which is a reliable and valid test for assessing manual dexterity [35] and is also considered a measure of functional ability was administered to each of the hands. Participants were requested to transfer as many blocks as they can, picking up one at a time, from one side of a box, over a divider, to the other side. The number of blocks transported from one side of the box to the other in 1 minute is recorded. The more blocks transferred in 1 minute indicates faster movements and better dexterity.

**Grip strength** of the affected (weaker) and the less-affected (stronger) hands was assessed using a Jamar Dynamometer in a standardized position [36] with the dynamometer handle on the second position. The mean of the three trials for each hand was recorded in kg. This test is a reliable and valid test for assessing manual grip strength [37, 38].

Daily use of the affected upper extremity (of a subgroup of the cohort) was assessed using the **Motor Activity Log (MAL)** [39]. This self-report questionnaire inquires about the amount and quality of the individual’s daily-use of their affected upper extremity for 14 daily activities such as picking up a glass or holding a book [39, 30]. The MAL has acceptable test-retest reliability and responsiveness [39, 40]. The MAL assesses actual use of the affected upper extremity in daily living and not only the functional ability (as assessed by using the ARAT and Box and Block Test).

In addition, independence in BADL was assessed using The Functional Independence Measure (FIM) [41] (administered as an interview [42]) and the IADL questionnaire [43, 44] assessed their independence in instrumental activities of daily living. Demographic and stroke information was also collected.

### Statistical analyses

Descriptive statistics were used to describe the participants in terms of stroke and demographic information, upper extremity measures and independence in daily living. Pearson correlations were used to assess the associations between the TLT to the upper extremity and other measures. Correlations ranging from 0.25 to 0.49 were considered fair, and values of 0.5 to 0.75 were considered moderate to good relationships [45]. Differences between groups were assessed using Independent-samples t-test for continuous variables and Chi square for dichotomous variables.

Cohen’s d Effect size (95% Confidence Interval) for Independent-samples t-test was based on calculating the mean difference between the two groups, and then dividing the result by the pooled standard deviation. Cohen’s d = 0.2 is considered a ‘small’ effect size, 0.5 represents a ‘medium’ effect size and 0.8 or larger is considered a ‘large’ effect size [46]. Bonferroni correction of significance level for multiple comparisons was used, therefore statistical significance was set at (α/n) 0.05/29 = p = 0.0017. All analyses were conducted using SPSS for Windows version 23.0 (SPSS Inc, Chicago, IL).

## Results

One-hundred and two individuals aged 29–85 years old with chronic stroke took part in this study. They were mean (SD) 20.9 (18.8) months post stroke onset and all lived at home in the community. Their demographic and stroke-related information appears in Table 1.

**Table 1:** Demographic and stroke related information of all participants and a comparison between participants with intact proprioception to participants with proprioception deficits.

	All participants (N = 102)	Proprioception intact N = 71	Proprioception deficits N = 31	Differences between groups
	Mean (SD)	Mean (SD)	Mean (SD)	t-test (p)
Age	59.6 (10.9)	58.8 (11.3)	61.5 (9.7)	-1.1 (.24)
Education	14.3 (5.5)	14.6 (6.4)	13.7 (2.8)	.7 (.45)
Months from stroke	20.9 (18.8)	19.0 (17.0)	25.2 (22.0)	-1.5 (.13)
Cognitive status—MMSE (0–30)	28.0 (2.1)	28.3 (2.1)	27.7 (2.2)	1.2 (.22)
Independence in BADL—FIM (18–126)*	108.3 (12.7)	112.5 (10.2)	98.8 (13.0)	5.7 (.00)
Independence in IADL—IADLq (0–23)**	14.2 (5.5)	15.7 (5.0)	10.6 (4.9)	4.7 (.00)
	N (%)	N (%)	N (%)	Chi square (p)
Sex M/F	69/33 (68/32)	46/25 (65/35)	23/8 (74/26)	.87 (.35)
Side of Stroke L/R	38/64 (37/63)	28/43 (39/61)	10/21 (32/68)	.47 (.49)
Dominant side effected Y/N	47/55 (46/54)	35/36 (49/51)	12/19 (39/61)	.60 (.42)

* FIM Effect size (95% confidence interval) - 1.23 (0.77–1.68)

** IADL Effect size (95% confidence interval) - 1.03 (0.57–1.46)

According to the TLT, 71 (69.6%) participants had intact proprioception, 25 (24.5%) participants had mild proprioception deficits and six (5.8%) participants had moderate proprioception deficits. Negative fair-moderate and statistically significant (p<.001) correlations were found between the severity of proprioception deficits (TLT) to the motor ability (FMA) (r = -.41), functional ability (ARAT) (r = -.48), dexterity (BBT) (r = -.43), grip strength (r = -.41) and daily-use (MAL amount and quality) (r = -.55 and r = -.54, respectively) of the affected upper extremity. Meaning that individuals with severe proprioception deficits demonstrated less motor and functional ability (or vice versa individuals with less motor and functional ability also demonstrated more severe proprioception deficits). A negative fair significant correlation was found between the scoring of TLT of the affected upper extremity with dexterity (r = -.23, p<.001) but not with grip strength of the less-affected (stronger) upper extremity. Note that correlations are negative due to the reverse scoring of the TLT; lower scores indicate better proprioception. The TLT was not significantly correlated (p>.05) with age, cognitive status (MMSE), side of stroke or time post-stroke.

For the further analysis, participants with mild and moderate proprioception deficits were grouped together to form a group of thirty-one (30.4%) participants with proprioception deficits. These 31 participants were compared to the 71 participants with intact proprioception.

No significant differences were found between groups in demographic (age, sex, years of education) or stroke related (months since onset, side of stroke, cognitive status) information (see Table 1). However, significant differences were found in independence in BADL and IADL; the group with proprioception deficits was less independent in BADL and IADL compared to the participants with intact proprioception (Table 1).

The affected upper extremity of the participants with intact proprioception had significantly more active movement, more functional ability and stronger grip strength compared to the participants with proprioception deficits (Table 2). Large values of effect size (1.01–1.50) were found for all of these measures (Table 2).

**Table 2:** Mean (standard deviation) of upper extremity measures of all participants and a comparison (Independent samples t-test and effect size) between participants with and without proprioception deficits.

		All participants (N = 102)	Proprioception intact N = 71	Proprioception deficits N = 31	Differences between groups t-test	Cohen’s d Effect size	Confidence Interval for Effect Size	
		Mean (SD)	Mean (SD)	Mean (SD)	t(100) (p)		Lower	Upper
Affected upper extremity	FMA (0–66)	39.1 (19.9)	45.1 (18.7)	25.4 (15.6)	5.1 (.00)	1.11	0.65	1.54
	ARAT (0–57)	34.5 (23.0)	41.5 (21.1)	18.4 (18.9)	5.2 (.00)	1.13	0.67	1.57
	Box & Block (#)	24.6 (21.3)	30.6 (20.4)	11.0 (16.9)	4.7 (.00)	1.01	0.56	1.45
	Grip strength (kg)	13.9 (11.4)	16.7 (11.8)	7.9 (6.8)	5.0 (.00)	1.30	0.83	1.75
	MAL amount* (1–5)	2.7 (1.8)	3.3 (1.6)	1.1 (1.1)	5.9 (.00)	1.50	1.02	1.96
	MAL quality* (1–5)	2.6 (1.6)	3.1 (1.4)	1.2 (1.1)	4.7 (.00)	1.44	0.97	1.90
Less-affected upper extremity	Box & Block (#)	51.1 (11.2)	53.0 (11.2)	46.7 (10.1)	2.7 (.01)	0.58	0.15	1.00
	Grip strength (kg)	30.0 (10.2)	29.9 (10.6)	30.1 (9.6)	-.1 (.93)	-0.02	-0.44	0.40

FMA-Fugl-Meyer Motor Assessment (motor ability); ARAT—Action Research Arm Test (functional ability); Box & Block Test (dexterity); MAL—Motor Activity Log (daily-use)

*N = 56—all participants (proprioception intact N = 41, proprioception deficit N = 15)

The less-affected (stronger) upper extremity of the group with proprioception deficits was not significantly different to the less-affected (stronger) upper extremity of the group with intact proprioception in terms of grip strength [29.9 (10.5) kg versus 30.1 (9.5) kg, respectively]. However, a significant difference (t(100) = 2.6, p<.01) with a medium effect size was found for the dexterity of the less-affected (stronger) hand between groups; participants with intact proprioception transferred more blocks with their less-affected (stronger) hand [52.9 (11.2) blocks] compared to the participants with proprioception deficits [46.6 (10.1) blocks]. This difference did not remain significant after the Bonferroni correction (p = .009).

## Discussion

The presence and impact of proprioception deficits at the chronic stage has not been the focus of many studies (e.g. Findlater [23]). Thirty percent of this cohort with chronic stroke had proprioception deficits, most participants experiencing mild deficits. This is lower than the prevalence reported [1, 47] and might be due to the assessment used in this study (TLT) or the inclusion criteria of the ability to walk at least 10-meters. This may have resulted in the exclusion of participants with severe proprioception deficits who are known not to regain their walking ability [4, 5].

The negative significant correlations found between the severity of proprioception deficits to the motor and functional ability, daily use, grip strength and dexterity of the affected upper extremity, demonstrate the associations (and not cause and effect) between the motor-functional ability and proprioception deficits. These associations of related constructs (convergent validity) together with no association of unrelated constructs, such as age and cognition (divergent validity) both support the construct validity of the TLT to assess proprioception [48]. Since sensory tests have limited validity, these findings are of importance.

Demographic characteristics, time since stroke, side of stroke or cognitive status were not different between groups. However, significant differences were found for the affected upper extremity in terms of motor, functional and grip strength between participants with and without proprioception deficits. These differences in motor and proprioception deficits are possibly due to the extended brain damage [4, 49, 50] in participants with proprioception deficits, resulting in worse upper extremity clinical symptoms.

These differences are perhaps not only from the initial brain damage caused by the stroke but also from the limited use of the upper extremity during the months since their stroke (as assessed by the MAL). Limited functional use of the upper extremity may have resulted in further restricted motor and functional ability. The effect size for differences between groups for the MAL (amount and quality measures) were the largest.

Interestingly, the differences between groups were not limited to the affected upper extremity. A medium effect size with a trend towards statistical significance regarding the difference in manual dexterity (but not grip strength) of the less-affected (stronger) upper extremity was found. It has been well established that the stroke affects the ipsilateral, as well as the contralateral hand, in terms of motor ability, reaction time, precision [51, 52] and even proprioception [53]. The dexterity of the less-affected (stronger) hand of all 102 participants [51.1 (11.2) blocks] is slower than the dexterity of healthy controls (mean age 73) [66.0 (9.1) blocks]. The association of proprioception deficits of the affected upper extremity and function of less-affected upper extremity should be further studied while taking into consideration hand dominance, which is also known to influence hand function [54].

The participants recruited for this study were overall independent in BADL since intact basic cognition and the ability to walk, were inclusion criteria. Nevertheless, the level of independence in BADL of the participants with intact proprioception was significantly higher than the participants with proprioception deficits (and a large effect size). A vast range of scores on the IADL questionnaire was found for both groups; 3–23 and 2–21 points/23 points for the participants without and with proprioception deficits. Yet, participants with intact proprioception were significantly more independent in IADL including shopping, use of transportation and cooking, compared to the participants with proprioception deficits (a large effect size). This difference in independence in IADL might be explained by the differences found between groups in mobility (the use of a walking aid 50% versus 80%) and function of both hands, which was higher for the participants with intact proprioception. In other words, better mobility and function of both hands of the participants with intact proprioception might have translated to more independence in IADL compared to the participants with proprioception deficits.

The main limitation of this is study is the fact that proprioception deficits were assessed solely on the Thumb Localization Test [5, 15]. Since the validity of the TLT, similar to other sensory tests, has not been well established [25], these findings should be carefully interpreted. The construct validity of the TLT, which was supported in this study, should be further researched. Possibly, by using additional ways of proprioception assessment or preferably by using advanced technology (such as robotic technology) [47], the prevalence and severity of proprioception deficits would be more accurate. Future research should include the assessment of other sensory modalities besides proprioception (using The Rivermead assessment of somatosensory performance [55, 56], which is a standardized tool). Other limitations of this study include the fact that due to the selection of participants (ability to walk at least 10-meters, live in the community, without a severe cognitive impairment), the percentage of individuals with proprioception deficits detected in this study might be lower than actually present in the population. In addition, these findings might not generalize to participants who are not able to ambulate or have cognitive decline.

To conclude, the presence of mild-moderate proprioception deficits of the affected upper extremity (as assessed by the TLT) is negatively associated with the motor, functional and daily-use of the affected upper extremity and dexterity of the less-affected upper extremity of individuals with chronic stroke. These participants are also less independent in BADL and IADL. Therefore, at the chronic stage, it is as important to assess proprioception deficits of individuals with stroke. Further studies should assess the effectiveness of treatments that focus on improving proprioception deficits as well as motor deficits to improve hand function and independence.

## Supporting information

S1 File PONE_proprioception data.sav. (SAV)
